# Perilesional edema in brain metastases: potential causes and implications for treatment with immune therapy

**DOI:** 10.1186/s40425-019-0684-z

**Published:** 2019-07-30

**Authors:** Thuy T. Tran, Amit Mahajan, Veronica L. Chiang, Sarah B. Goldberg, Don X. Nguyen, Lucia B. Jilaveanu, Harriet M. Kluger

**Affiliations:** 1 Yale School of Medicine and Yale Cancer Center, Yale University, New Haven, CT USA; 2 Yale School of Medicine and Yale Department of Radiology & Biomedical Imaging, Yale University, New Haven, CT USA; 3 Yale School of Medicine and Yale Department of Pathology, Yale University, New Haven, CT USA; 4 Yale School of Medicine and Yale Department of Neurosurgery, Yale University, New Haven, CT USA

**Keywords:** Melanoma, Non-small cell lung cancer, Metastasis, Blood-brain barrier, Edema

## Abstract

**Background:**

Little is known about tumor-associated vasogenic edema in brain metastasis, yet it causes significant morbidity and mortality. Our purpose was to characterize edema in patients treated with anti-PD-1 and to study potential causes of vessel leakage in humans and in pre-clinical models.

**Methods:**

We analyzed tumor and edema volume in 18 non-small cell lung (NSCLC) and 18 melanoma patients with untreated brain metastases treated with pembrolizumab on a phase II clinical trial. Melanoma brain metastases were stained with anti-CD34 to assess vessel density and its association with edema. We employed an in vitro model of the blood-brain barrier using short-term cultures from melanoma brain and extracranial metastases to determine tight junction resistance as a measure of vessel leakiness.

**Results:**

Edema volumes are similar in NSCLC and melanoma brain metastases. While larger tumors tended to have more edema, the correlation was weak (*R*^2^ = 0.30). Patients responding to pembrolizumab had concurrent shrinkage of edema volume and vice versa (*R*^2^ = 0.81). Vessel density was independent of the degree of edema (*R*^2^ = 0.037). Melanoma brain metastasis cells in culture caused loss of tight junction resistance in an in vitro blood-brain barrier model system in some cases, whereas extracerebral cell cultures did not.

**Conclusions:**

Edema itself should not preclude using anti-PD-1 with caution, as sensitive tumors have resultant decreases in edema, and anti-PD-1 itself does not exacerbate edema in sensitive tumors. Additional factors aside from tumor mass effect and vessel density cause perilesional edema. Melanoma cells themselves can cause decline in tight junction resistance in a system void of immune cells, suggesting they secrete factors that cause leakiness, which might be harnessed for pharmacologic targeting in patients with significant perilesional edema.

**Electronic supplementary material:**

The online version of this article (10.1186/s40425-019-0684-z) contains supplementary material, which is available to authorized users.

## Background

Brain metastases (BMs) are the most common intracerebral malignancies in adults; melanoma has the highest propensity for brain dissemination, followed by non-small cell lung cancer (NSCLC) [1]. Intracranial disease control by surgery or radiation has been the mainstay of treatment. Immune checkpoint inhibitors (CPIs) have provided significant benefit in treating advanced melanoma and NSCLC. Monoclonal antibodies to CTLA-4, PD-1, and PD-L1 have been approved for advanced melanoma and/or NSCLC [2]. Due to concerns about drug penetration across the blood-brain barrier (BBB), initial trials using CPIs excluded patients with untreated BMs, and activity of CPIs in BMs was unknown until recently. Promising phase II trials now show activity and acceptable neurologic toxicity using immune therapy in untreated melanoma or NSCLC BMs [3–7].

Neurologic symptoms from BMs are often caused by edema rather than from the tumor itself, as edema volume can be several-fold greater than tumor volume. Corticosteroids remain the primary treatment for symptomatic edema, but higher doses may render CPIs ineffective and produce side effects [5, 8]. While progress has been made towards understanding activity of contemporary drugs in BMs, research is still needed to determine how best to treat patients with neurological symptoms, patients requiring corticosteroids, and patients with significant perilesional edema. CPI effects on edema are understudied, as edema is often not recorded or quantitated in clinical trials. Little is known about the relationship between tumor-associated vasogenic edema and tumor volume or survival [9].

We sought to address these issues by evaluating tumor and edema volumes in a cohort of 18 NSCLC and 18 melanoma patients with untreated BMs enrolled on a phase II trial using pembrolizumab [4]. We hypothesized that larger tumors have more associated perilesional edema, but this finding has never been verified objectively given technical difficulties with accurate edema quantitation due to irregular borders and overlap with nearby tumors.

Defects in BBB inter-endothelial tight junctions are thought to cause peritumoral edema [10, 11]. Little is known about the cell types that cause edema and how CPIs affect edema. We examined blood vessel density in brain metastases and employed short-term cultures of patient-derived melanoma BM cells to model the BBB in vitro and evaluate endothelial barrier function and the effects of melanoma cells on tight junction resistance.

## Methods

### Brain metastasis patients treated with pembrolizumab

We retrospectively analyzed 18 NSCLC and 18 melanoma patients at Yale Cancer Center enrolled in a phase 2 trial of pembrolizumab (10 mg/kg, IV every 2 weeks) for untreated BMs, NCT02085070 [4, 12]. Patients were ineligible if they had symptomatic edema requiring corticosteroids or intracranial metastasis > 2 cm not previously irradiated. BM response was evaluated by MRI using modified RECIST 1.1 every 8 weeks.

### Tumor and edema volume quantitation

Enrollment MRIs were analyzed using 3D Slicer (https://www.slicer.org) [13–15]. Images were segmented using 3D T1-weighted MP-RAGE or FLAIR sequences. We applied the Fast GrowCut Extension with Laplacian 0 settings to generate 3D models with corresponding tumor (V_T_) and edema (V_E_) volumes. To normalize edema to tumor volumes, (edema:tumor) ratios were calculated as (V_E_)/(V_T_). Up to five of the largest tumors per patient were evaluated. Tumors were excluded if nearby lesions caused overlapping edema or if images could not be processed by 3D Slicer.

### Vessel density by CD34 staining on cerebral metastatic melanoma samples

A previously constructed and characterized tissue microarray containing formalin-fixed, paraffin-embedded tumor tissue from 30 melanoma BM patients was stained with anti-CD34 (Dako) to determine micro-vessel density, which was quantitated as detailed previously (Additional file 1: Supplemental Methods) [16, 17].

### In vitro blood-brain barrier assay

Short-term melanoma cultures were obtained from resected MBMs, used within 20 passages, and denoted by the prefix “YU” for Yale University (Additional file 2: Table S1). A375P and its cerebrotropic daughter cell line A375Br were a gift from Dr. Huang [18]. Co-cultures of primary human umbilical vein endothelial cells (HUVEC, ScienCell) and primary human astrocytes (ScienCell) recapitulating the BBB were established, as previously published and detailed in Additional file 1: Supplemental Methods [19]. Establishment of the BBB was confirmed via expression of brain endothelial markers glucose transporter-1 (GLUT1) and γ-glutamyl transpeptidase (GGT1) (Additional file 3: Figure S1) [20]. BBB leakiness was assessed by changes in transendothelial electrical resistance (TEER) and confirmed via lack of Evans Blue-labeled albumin (0.45% in phenol red-free medium) passage after 30 min at 37 °C using a previously established protocol (Additional file 4: Figure S2) [19, 21].

### Statistical analysis

Correlations were analyzed by the coefficient of determination. Clinical variables were compared by unpaired t-tests. Progression free survival (PFS) and overall survival (OS) analyses were depicted by the Kaplan–Meier method and stratified by log-rank test. Comparison of in vitro TEER to in vivo edema was done by Fisher’s exact test.

## Results

### Tumor and edema volumes in NSCLC or melanoma patients are weakly correlated

**Fig. 1:**
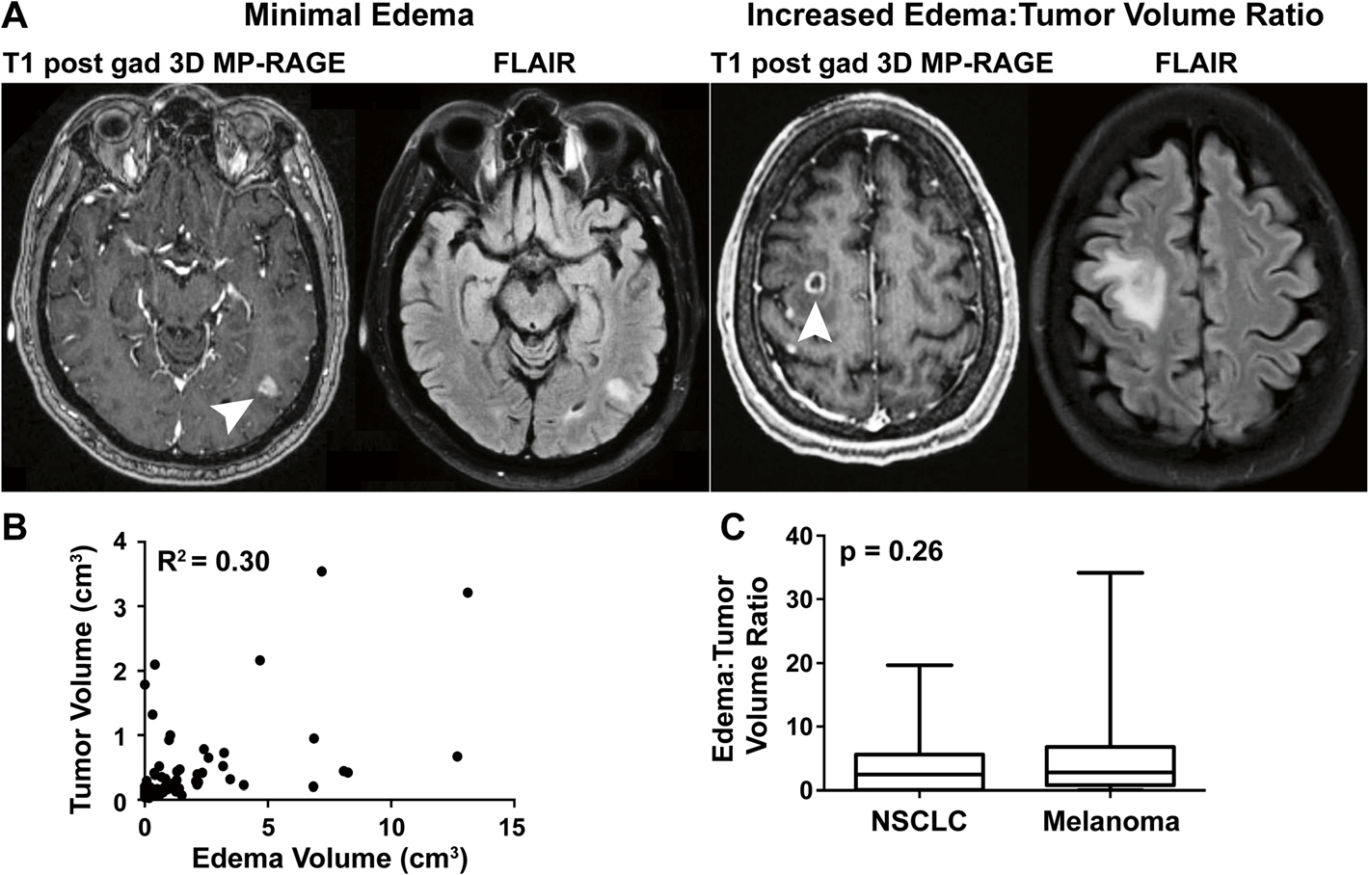
Tumor volume weakly correlates with edema among NSCLC and melanoma patients. **a** Representative baseline MRIs taken from patients treated with pembrolizumab showing similar tumor volumes on T1 post gadolinium 3D MP-RAGE (white arrows) but discordant amounts of edema volume on FLAIR sequences. **b** The correlation between tumor and edema volume of NSCLC and melanoma BMs is weak. **c** Pre-treatment edema:tumor volumes are not significantly different in melanoma and NSCLC patients

Based on clinical observations that BMs of similar size can induce variable edema volumes (examples shown in Fig. 1a), we used 3D modeling to quantitate tumor and edema volumes. The correlation between tumor and edema volumes prior to anti-PD-1 was weak (*R*^2^ = 0.30, *p* < 0.0001, Fig. 1b). While melanoma BMs tended to be more edematous, this observation did not reach statistical significance (*P* = 0.059, Additional file 5: Figure S3A). Given the weak relationship between size and edema, we assessed edema:tumor volume ratios, and these were similar in NSCLC and melanoma both when comparing all tracked BMs (*P* = 0.26, Fig. 1c) or the largest lesion per patient (*P* = 0.67, Additional file 5: Figure S3B). Edema:tumor volumes by NSCLC subtype (adenocarcinoma, squamous cell, or poorly differentiated carcinoma) (*P* > 0.05, data not shown) or by lactate dehydrogenase level in melanoma patients (*P* = 0.83, data not shown) were also similar. Patient characteristics were previously described [4].

### Response to anti-PD-1 was independent of baseline edema

**Fig. 2:**
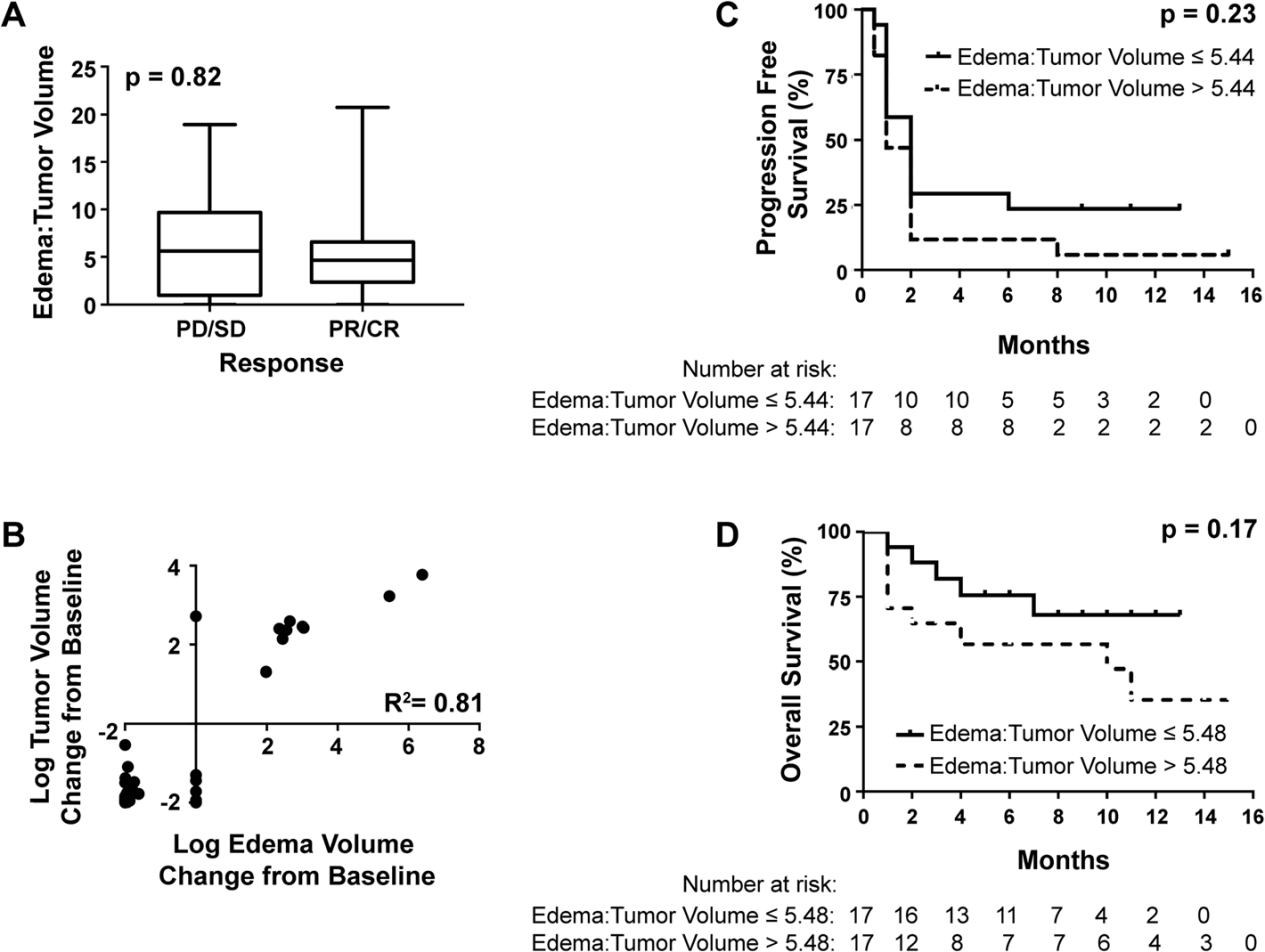
Pembrolizumab response and survival were independent of initial edema:tumor volumes and concordant with edema change. **a** Pre-treatment BM edema:tumor volume ratio does not impact response to pembrolizumab (PD-progression of disease, SD-stable disease, PR-partial response, and CR-complete response, by modified RECIST version 1.1). **b** Tumor and edema volume changes from baseline after 4 cycles of pembrolizumab were strongly correlated. As pembrolizumab-sensitive BMs shrank, the tumor-associated edema also shrank and vice-versa for pembrolizumab-resistant tumors. Due to the wide range in values, the log percent change was used for graphical presentation. **c** Kaplan-Meier survival curves show no significant difference between BM PFS in patients whose edema:tumor volume was above the median compared to those whose ratio was below the median; **d** overall survival of NSCLC and melanoma patients was similarly not affected by baseline edema:tumor volume ratios

As there was no difference in edema volumes between melanoma and NSCLC patients when correcting for tumor size, we combined the cohorts for subsequent analyses. Historically, there has been hesitancy to treat BMs with CPIs due to concern for edema exacerbation, but we found that pre-treatment edema:tumor volume did not impact the likelihood of response to pembrolizumab in the brain (*P* = 0.82, *N* = 14 evaluable NSCLC and 13 melanoma patients, Fig. 2a).

### Changes in tumor and edema volume with anti-PD-1 are concordant

Changes in edema and tumor volume after 4 cycles of pembrolizumab compared with baseline were strongly correlated (*R*^2^ = 0.81, *N* = 11 NSCLC and 8 melanoma patients with 27 and 17 target lesions, respectively, as 3 NSCLC and 5 melanoma patients were excluded due to disease progression or immune-related side effects prior to receiving 4 cycles of treatment, Fig. 2b). Patients who responded to therapy had corresponding decreases in tumor and edema volume and vice versa.

### Progression-free and overall survival are independent of baseline edema in pembrolizumab-treated patients

To determine whether more edematous lesions were associated with worse PFS or OS, we binarized edema:tumor volume ratios by the median. There was no statistically significant difference between low and high edema:tumor ratios and PFS (*P* = 0.23, Fig. 2c). There was a trend towards an association between improved OS and lower edema:tumor ratios, but this was not significant (*P* = 0.17, Fig. 2d).

### Peritumoral edema is not correlated with vessel density in human melanoma brain metastases

**Fig. 3:**
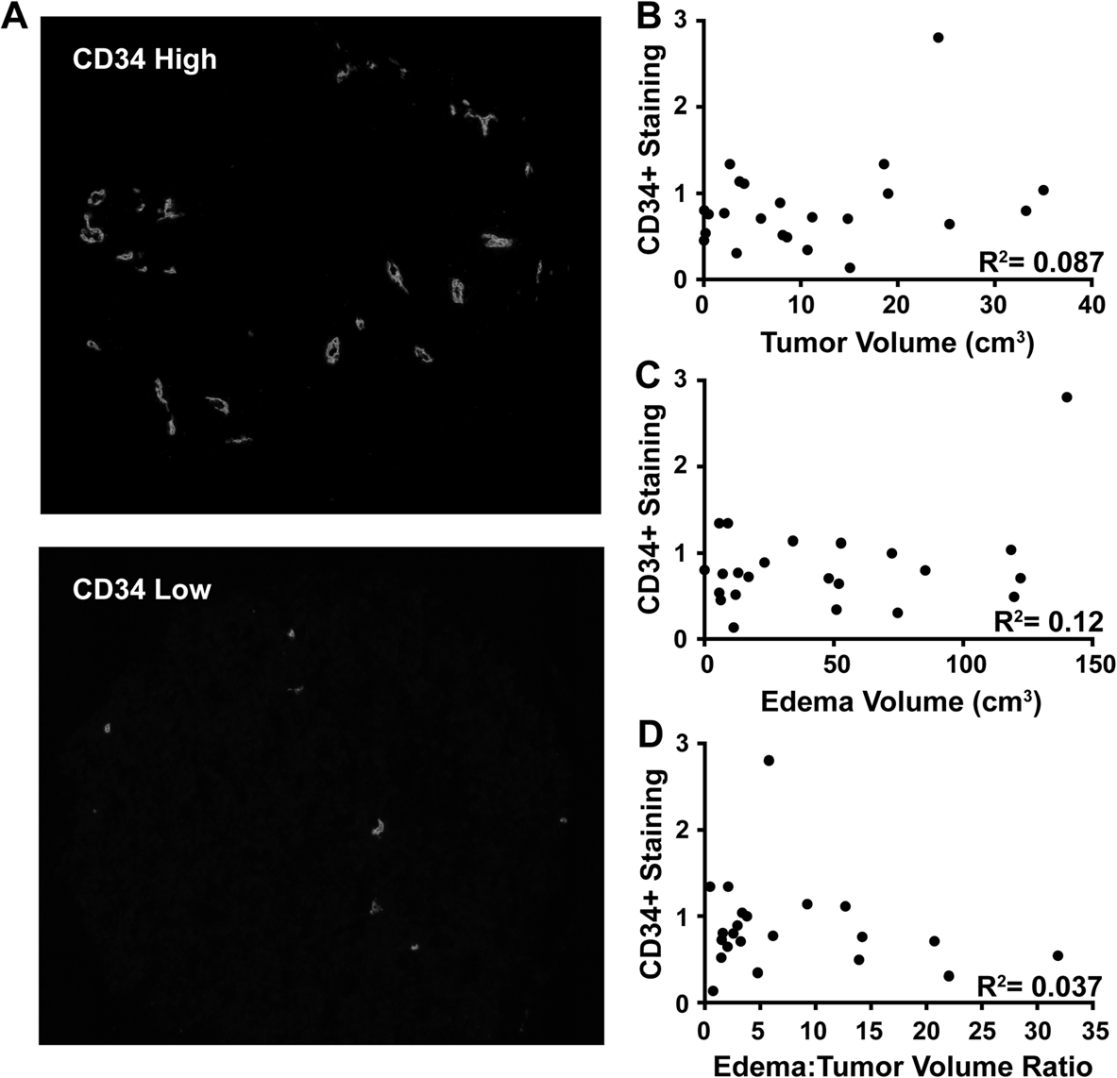
Edema is not correlated with micro-vessel density. **a** Representative images of melanoma BM core sections with either high or low anti-CD34^+^ staining. There were no correlations between density of CD34^+^ staining cells in melanoma BMs and pre-resection **b** tumor volume, **c** edema volume, or **d** edema:tumor ratio

One potential mechanism of edema is increased tumor neo-angiogenesis resulting in formation of functionally aberrant, leaky neo-vessels. Tumor vessel density was determined in 23 melanoma craniotomy samples with available pre-operative MRIs; similar samples were not available for NSCLC patients. There was no correlation between vessel density and either tumor volume (*R*^2^ = 0.087), edema volume (*R*^2^ = 0.12), or edema:tumor ratio on pre-resection MRI (*R*^2^ = 0.037, Fig. 3a-d).

### Cultured melanoma cells from cerebral metastases may induce BBB leakiness, whereas cells from extra-cerebral metastases do not

**Fig. 4:**
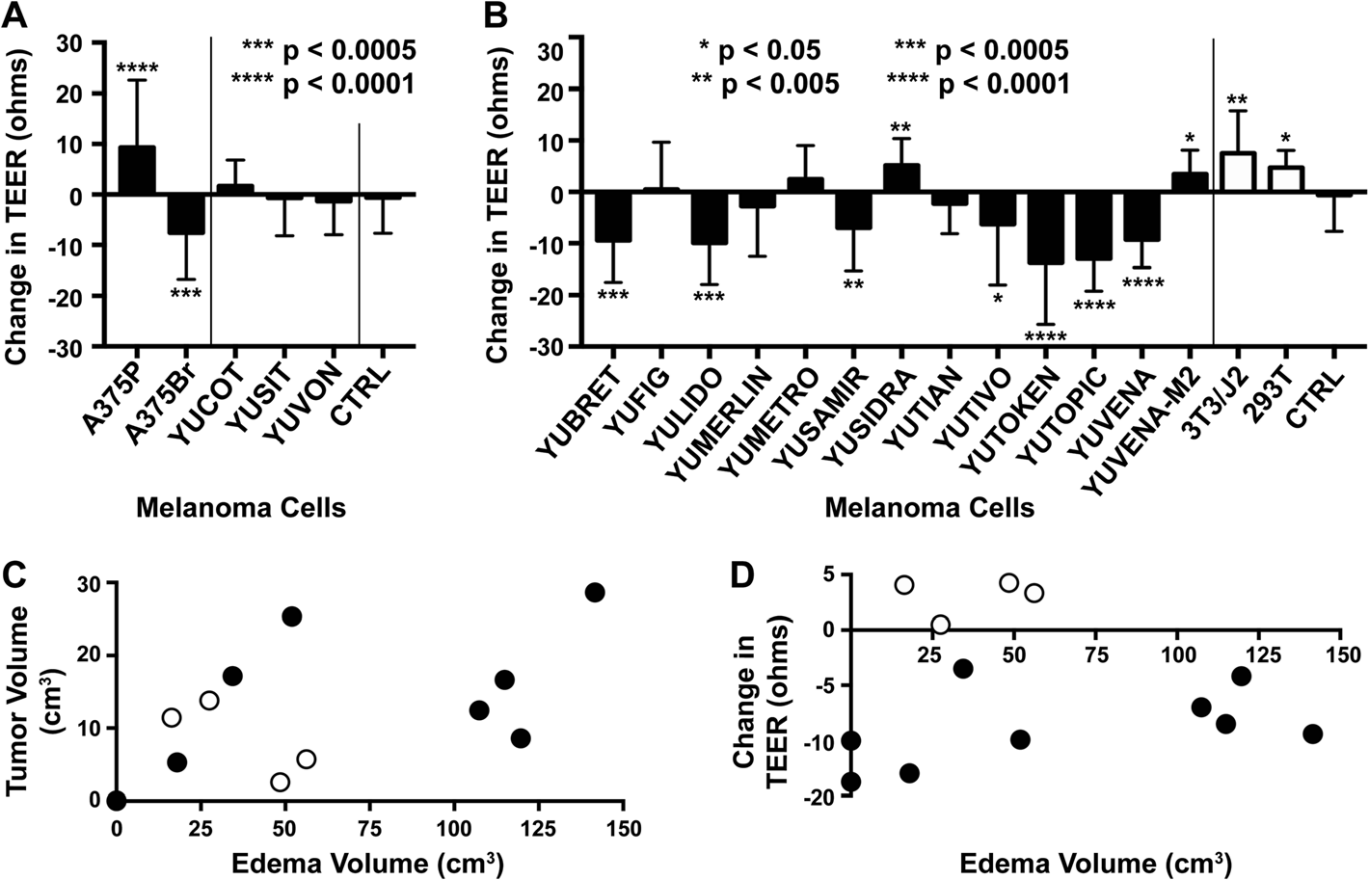
Specific human melanoma BM cells induce BBB compromise without contributions of immune cells in vitro. **a** TEER was measured as a surrogate for inter-endothelial tight junction integrity in an in vitro BBB model. A significant decrease in TEER was found in a cerebrotropic human melanoma daughter cell line (A375Br), derived by repeated intra-carotid injection of A375P parental cells and expansion of cells isolated from the brain. A375P cells derived from a metastatic lymph node increased TEER. Short-term melanoma cultures from other extracerebral sites (YUCOT, YUSIT, and YUVON) similarly did not result in decreased resistance compared to CTRL (HUVEC and astrocytes co-cultured without the addition of melanoma cells). **b** A panel of short-term cultures derived from human melanoma BMs was similarly studied and TEER changes determined. Co-culture with some but not all melanoma BMs resulted in decreased resistance compared to CTRL. **c** TEER changes were compared to MRI-based tumor and edema volume measurements of resected lesions from which the cultures were derived. Black circles represent cell lines with decreased TEER, whereas white circles correspond to cell lines with increased TEER changes*.* **d** Four of the nine cell cultures that decreased TEER (black circles) are from more edematous tumors, whereas all the cell cultures that did not decrease TEER (white circles) were from less edematous tumors

To determine the effects of tumor cells themselves on edema, we first established TEER as a surrogate for BBB leakiness by demonstrating that passage of albumin strongly correlated with decreased TEER (*R* = -0.69, *P* = 0.0045, Additional file 4: Figure S2). As vasogenic edema is uniquely associated with cerebral metastases, we determined whether cells cultured from extra-cerebral metastases resulted in decreased TEER when co-cultured with HUVEC cells and astrocytes in our BBB model. Co-cultures with A375P cells (derived from a lymph node) resulted in increased TEER (*P* < 0.0001 compared to CTRL containing only HUVECs and astrocytes), whereas its cerebrotropic daughter cell line A375Br resulted in decreased TEER (*P* = 0.0005) [18]. Further testing with other extracerebral cultures YUCOT, YUSIT, and YUVON also did not result in decreased TEER (*P* > 0.05, Fig. 4a). To assess how intracerebral metastases affect the BBB, 13 short-term cultures of human melanoma BMs were assayed using the in vitro BBB model. Similar NSCLC cultures were not available. TEER decreases were observed after the addition of 9 of the 13 cultures (Fig. 4b), compared to CTRL or to 293 T human embryonic kidney and 3T3/J2 murine fibroblast cell lines. These results are consistent with the clinical picture in which not all cerebral metastases are associated with edema, and perilesional edema is not typically observed in other organs.

TEER changes were then compared to edema:tumor ratios on matched-patient, pre-resection MRIs. Melanoma BMs with edema >75cm^3^ or tumors >20cm^3^ had corresponding TEER decreases in vitro (black circles, Fig. 4c). Smaller tumors and those with less edema on pre-resection MRI could have either decreased (black circles) or increased TEER changes (white circles) from CTRL, demonstrating that the in vitro TEER data matches in vivo human data in some, but not all cases (*P* = 0.59, Fig. 4d). This finding indicates that additional factors related to the host or the tumor microenvironment also affect tight junction resistance.

## Discussion

Brain metastatic disease presents an ongoing clinical challenge in patients with advanced malignancies, partly due to neurologic symptoms arising from tumor-associated vasogenic edema. As 3D imaging technology improves, we are now able to study the relationship between cerebral tumor and edema volume. There historically has been reluctance to administer CPIs in individuals with untreated BM primarily due to questionable intracranial activity. However, additional concerns include increasing peritumoral inflammation and vasogenic edema. Furthermore, corticosteroids required to treat symptomatic peritumoral edema may impede CPI activity, although a recent retrospective study in lung cancer suggests that this might be due to worse underlying prognosis in patients receiving steroids rather than antagonistic drug effects [5, 22]. To the best of our knowledge, we are the first to evaluate edema in the context of CPI use in untreated BMs. We found that NSCLC and melanoma BMs behave similarly—in both tumor types larger lesions tend to have more edema, possibly due to mechanical obstruction of venous drainage, but the correlation is weak. There were no differences in edema:tumor volume ratios between the two cancer types, in different NSCLC histologic subtypes, or in melanomas with high LDH. When comparing changes in tumor and edema volumes before anti-PD-1 and after 4 cycles, cerebral edema and tumor volumes responded concordantly. We therefore conclude that in asymptomatic patients with lesions up to 2 cm, the edema is unlikely to worsen when tumors are anti-PD-1 sensitive. Importantly, all patients must be monitored by a multidisciplinary team for signs and symptoms of worsening edema, particularly as they likely indicate resistance to anti-PD-1. Furthermore, we evaluated whether baseline edema provided prognostic or predictive significance and found no statistically significant association between edema and PFS or OS in patients treated with pembrolizumab, but further studies with larger sample sizes are required to validate this observation.

Factors beyond tumor size that might cause edema include an abundance of neo-vessels and secretion of factors from tumor or immune cells that disrupt the BBB. We found no association between edema and micro-vessel density by CD34 staining of tumor-associated blood vessels. We conclude that edema is mediated through a functional rather than quantitative difference in tumor-associated blood vessels. This effect could be due to melanoma cells or their interaction with the brain tumor microenvironment, consisting of immune cells or neurovascular supporting cells. Several factors have already been implicated in peritumoral edema in other cancers, such as vascular endothelial growth factor-A, aquaporin-4, and metalloproteinases, but further study is needed to identify the critical edema mediators in melanoma and to understand how they are affected by CPIs [23].

To determine the effects of melanoma cells on tight junction resistance, we used an in vitro BBB model devoid of immune cells. Cultures from extra-cerebral sites did not result in decreased TEER, which we established as a surrogate for vessel leakiness, whereas nine of 13 cultures from BMs were associated with decreased TEER. When comparing TEER changes to edema volume on pre-craniotomy MRI, we found that all of the more edematous tumors (>75cm^3^) were associated with decreased TEER on the in vitro assay, whereas cell cultures from less edematous tumors (<75cm^3^) on MRI had variable effects on TEER.

Thus, only some melanoma cells secrete factors that affect tight junction resistance, and there are likely more complex interplays between the tumor and its microenvironment, such as with astrocytes, pericytes, microglia, and neurons that maintain BBB homeostasis, and inflammatory cells. Endothelial monolayer disruption and leakiness could be the result of melanoma-secreted factors, such as by serine protease, matrix metalloproteinase, or cytokines [24]. Elucidating the mechanism by which melanoma cells, the immune system, or their interplay induce vasogenic edema will be the focus of future studies. To our knowledge, this is the first report correlating patient in vivo edema response to matched in vitro BBB behavior. Our future studies will focus on co-culture systems with autologous T cells and establishing an in vivo model of tumor-associated vasogenic edema using patient xenografts. NCT02681549 and NCT03175432 are ongoing trials evaluating PD-1 or PD-L1 in combination with bevacizumab, an anti-vascular endothelial growth factor therapy, in untreated BMs to determine whether modulation of the microvascular BBB environment can enhance CPI effectiveness. It will be interesting to evaluate these combinations for synergistic edema responses in these patients, given the use of bevacizumab to treat steroid-refractory vasogenic edema.

To understand whether the ability to mediate edema is acquired as tumor cells home to the brain, we tested 4 extracerebral metastatic human melanoma short-term cultures, A375P, YUCOT, YUSIT, and YUVON, in the in vitro BBB. All extracerebral metastases did not decrease TEER. Interestingly, the cerebrotropic daughter line A375Br, which was derived through serial injection and expansion of A375P BMs, could induce a significant decrease in TEER. This finding could indicate an acquired ability to breakdown the BBB that developed during cerebrotropism. However, the mechanism remains to be elucidated and is a focus of ongoing animal studies.

## Conclusions

Tumor-associated vasogenic edema presents a significant challenge in treating patients with metastatic brain disease. In brain metastasis patients treated with anti-PD-1, the extent of pre-treatment edema should not be a barrier to anti-PD-1 therapy in asymptomatic patients with small BMs. Anti-PD-1 sensitive tumors had resultant decreases in edema and tumor, and PFS and OS were not associated with the degree of baseline edema. In some but not all cases, melanoma cells can directly induce BBB disruption in vitro in a model system void of immune cells. Identifying the mechanisms by which tumor cells, immune cells, or their interplay, induce BBB breakdown is vital for developing therapies that modulate edema without inhibiting immune function.

## Additional files

Additional file 1: Supplemental Methods. (DOCX 16 kb)

Additional file 2: **Table S1.** Demographic, anatomic, prior treatment, and mutational information from patient-derived, short-term melanoma cell cultures. (DOCX 1304 kb)

Additional file 3: **Figure S1.** Recapitulation of the in vitro BBB in endothelial and astrocyte co-cultures. (DOCX 2394 kb)

Additional file 4: **Figure S2.** Quantitation of leakiness in the in vitro BBB assay and correlation with TEER results. (DOCX 2072 kb)

Additional file 5: **Figure S3.** NSCLC and melanoma brain metastases have similar degrees of perilesional edema. (DOCX 1306 kb)

## Data Availability

Data sharing is not applicable to this article as no datasets were generated or analysed during the current study.

## Abbreviations

3D: 3-dimensional
BBB: Blood-brain barrier
BMs: Brain metastases
CPIs: Checkpoint inhibitors
NSCLC: Non-small cell lung cancer
OS: Overall survival
PFS: Progression free survival
TEER: Transendothelial electrical resistance
