# Supplementary material for: Perilesional edema in brain metastases: potential causes and implications for treatment with immune therapy
Source: J Immunother Cancer. 2019 Jul 30;7:200. doi: 10.1186/s40425-019-0684-z (PMC6668163; doi:10.1186/s40425-019-0684-z)
Supplement: Supplementary file 1 — Supplemental Methods. (DOCX 16 kb) [file 40425_2019_684_MOESM1_ESM.docx]

Additional file 1 Supplementary Methods

**Vessel Density by CD34 Staining on Cerebral Metastatic Melanoma Samples**

All tissue underwent formal central review by a pathologist who identified representative tumor regions for inclusion and excluded areas of necrosis or hemorrhage. Three 0.6mm cores from each tumor in 5μm sections were subjected to de-paraffinization, sodium citrate antigen retrieval, and incubation with primary antibody diluted 1:50 in 0.3% BSA/1X TBS overnight at 4°C followed by an anti-mouse horseradish peroxidase polymer backbone (Envision, Dako), and Cy5-tyramide (PerkinElmer Life Science Products). The tumor mask was generated using rabbit S100 (1:100 dilution, Dako) to identify melanoma cells followed by anti-rabbit secondary goat IgG conjugated to Alexa-546 (Molecular Probes). Slides were mounted with ProLong Gold containing 4′,6-Diamidino-2-phenylindole (DAPI, Invitrogen) to permit nuclei visualization. Micro-vessel density scores were calculated by dividing the area of CD34, Cy5 positivity by the histospot area. Core tissue containing <3% of tumor was excluded from analysis.

***In vitro* Blood-Brain Barrier Assay**

Melanoma cells were grown in Opti-MEM I (Gibco) supplemented with 5% heat-inactivated fetal bovine serum (Sigma) and 1% antibiotic-antimycotic 100X (Gibco). 5x10^4^ HUVECs were co-cultured with 10^5^ primary human astrocytes on the apical and basal membranes, respectively, of 3µm pore PET tissue culture inserts (Fisher) which had been previously coated for 30 minutes with 0.2% gelatin on the apical membrane (Sigma) and overnight with 1µg mL^-1^ polylysine on the basal membrane (ScienCell). Transwells were co-cultured for 3 days in phenol-red free endothelial cell medium (ScienCell) followed by transendothelial electrical resistance (TEER) measurement using STX2 chopstick electrodes attached to an EVOM^2^ meter (World Precision Instruments). Subsequently, 5x10^4^ melanoma cells were added to the apical membrane, and TEER was remeasured after 24 hours. Controls included HUVEC/astrocyte co-cultures without melanoma cells (CTRL), insert alone, and HUVEC/astrocyte co-cultures with murine fibroblast 3T3/J2 cells (Kerafast) or human embryonic kidney 293T cells (ATCC). Corrected TEER values were calculated by subtracting the average TEER of coated inserts without cells from the TEER of experimental samples; this value was then subtracted from the baseline TEER measured on the same transwell immediately prior to addition of melanoma cells. Finally, all values were normalized to CTRL for graphical presentation. BBB markers GLUT1 and GGT1 were quantified using qPCR using TaqMan gene expression assays (Applied Biosystems) after RNA extraction or by Western protein quantification from CTRL and transwells containing HUVECs without astrocytes (Qiagen).
