# Supplementary material for: Perilesional edema in brain metastases: potential causes and implications for treatment with immune therapy
Source: J Immunother Cancer. 2019 Jul 30;7:200. doi: 10.1186/s40425-019-0684-z (PMC6668163; doi:10.1186/s40425-019-0684-z)
Supplement: Supplementary file 2 — Table S1. Demographic, anatomic, prior treatment, and mutational information from patient-derived, short-term melanoma cell cultures. (DOCX 1304 kb) [file 40425_2019_684_MOESM2_ESM.docx]

Additional file 2 **Table S1** Demographic, anatomic, prior treatment, and mutational information from patient-derived, short-term melanoma cell cultures. Cells originated from patients with a mean age of 62 years ±10 standard deviation, 80% were male, 44% were BRAF mutated, 25% were NRAS mutated, and of those derived from brain metastases, 15% had steroids within 24 hours prior to pre-resection MRI, and 46% had ≥1 prior systemic therapy. Mutational status did not impact TEER changes in the *in vitro* BBB model (*P*>0.99, Fisher’s exact test).

**
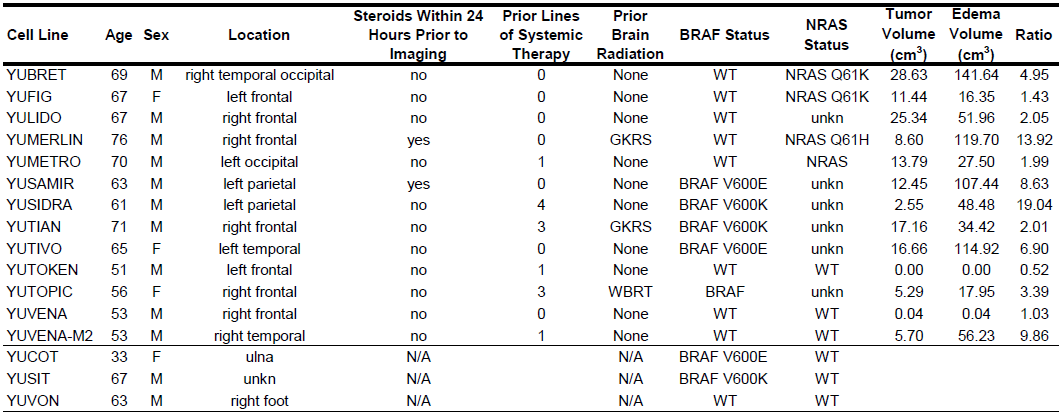
**
