# Supplementary material for: Perilesional edema in brain metastases: potential causes and implications for treatment with immune therapy
Source: J Immunother Cancer. 2019 Jul 30;7:200. doi: 10.1186/s40425-019-0684-z (PMC6668163; doi:10.1186/s40425-019-0684-z)
Supplement: Supplementary file 3 — Figure S1. Recapitulation of the in vitro BBB in endothelial and astrocyte co-cultures. (DOCX 2394 kb) [file 40425_2019_684_MOESM3_ESM.docx]

Additional file 3 **Figure S1** Recapitulation of the *in vitro* BBB in endothelial and astrocyte co-cultures. **(A)** GLUT1 mRNA expression showing upregulation of GLUT1 in HUVEC/Astrocyte co-cultures compared to either HUVECs alone or human brain microvascular endothelial cells (HMBECs)/Astrocytes or HMBECs alone. **(B)** GGT1 protein expression showing relatively increased GGT1 in HUVEC/Astrocyte co-cultures compared to HUVECs alone.

**
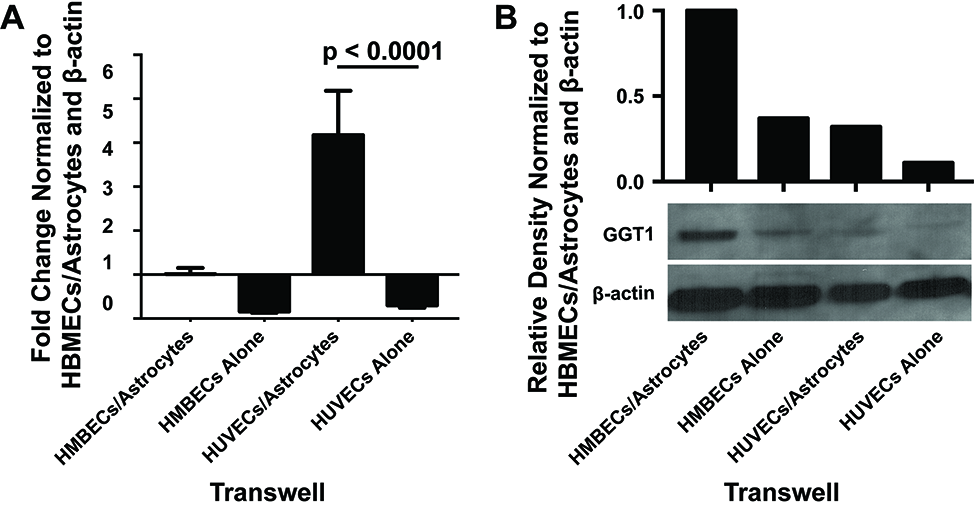
**
