# Supplementary material for: Perilesional edema in brain metastases: potential causes and implications for treatment with immune therapy
Source: J Immunother Cancer. 2019 Jul 30;7:200. doi: 10.1186/s40425-019-0684-z (PMC6668163; doi:10.1186/s40425-019-0684-z)
Supplement: Supplementary file 4 — Figure S2. Quantitation of leakiness in the in vitro BBB assay and correlation with TEER results. (DOCX 2072 kb) [file 40425_2019_684_MOESM4_ESM.docx]

Additional file 4 **Figure S2** Quantitation of leakiness in the *in vitro* BBB assay and correlation with TEER results. **(A)** To confirm validity of TEER results, Evans Blue-labeled albumin was added to the luminal transwell chamber, and quantity of dye was measured in the abluminal compartment after 30 minutes. **(B)** A strong correlation existed between TEER change and relative albumin permeability.

**
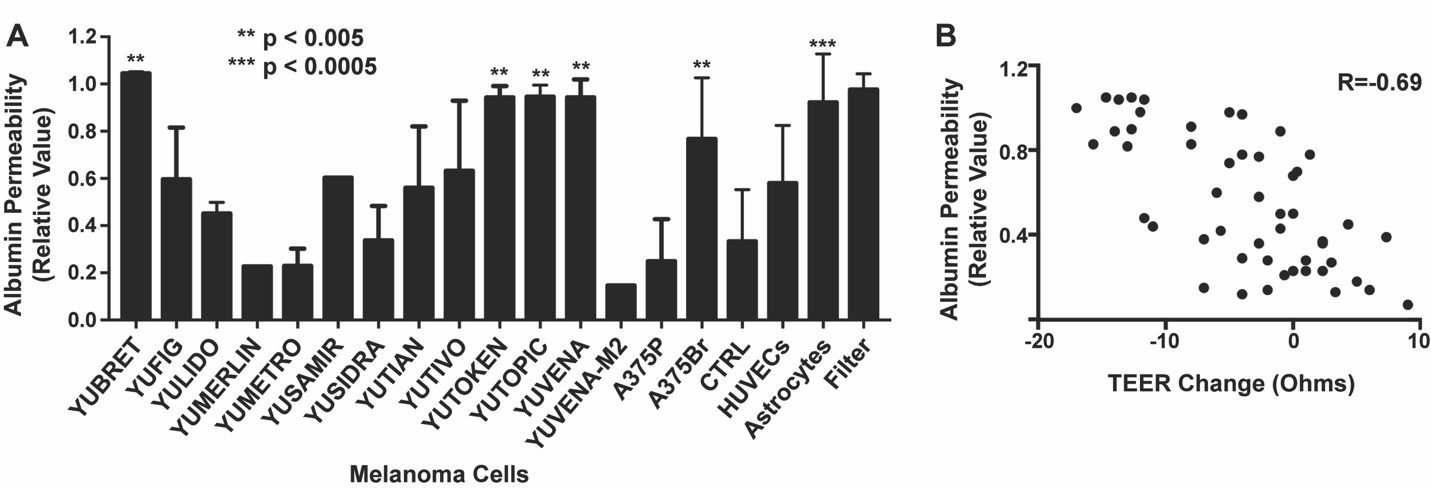
**
