# Supplementary material for: Perilesional edema in brain metastases: potential causes and implications for treatment with immune therapy
Source: J Immunother Cancer. 2019 Jul 30;7:200. doi: 10.1186/s40425-019-0684-z (PMC6668163; doi:10.1186/s40425-019-0684-z)

Additional file 5 **Figure S3** NSCLC and melanoma brain metastasis have similar degrees of perilesional edema. **(A)** No differences were observed in edema volume of all monitored brain lesions. **(B)** Edema:tumor volume ratios of the largest tracked brain lesion per study participant were similar in NSCLC and melanoma patients at trial enrollment (6.41 ±1.26 standard of the mean [SEM] in NSCLC versus 7.54 ±2.37 SEM in melanoma brain metastasis patients).


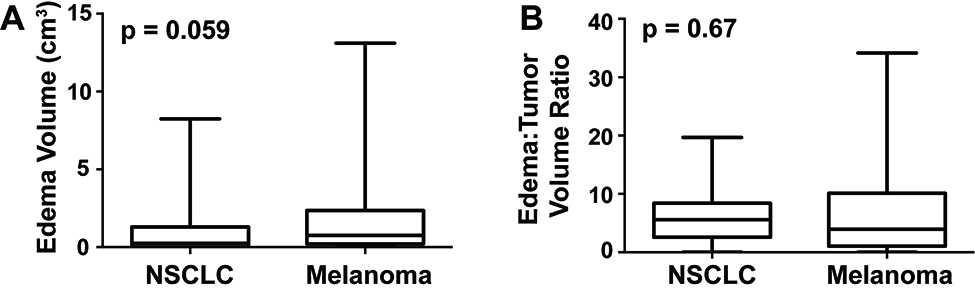

Supplement: Supplementary file 5 — Figure S3. NSCLC and melanoma brain metastases have similar degrees of perilesional edema. (DOCX 1306 kb) [file 40425_2019_684_MOESM5_ESM.docx]
